# Prevention of Bad Teeth

**Published:** 1886-02

**Authors:** 


### Prevention of Bad Teeth.

A foreign exchange says the troubles which arise from disease of the teeth, or from their loss, are not always directly referable to their cause. When actual toothache is present there is of course little doubt, and the remedy of extraction at once presents itself. At the same time, it must be remembered that the forceps does not undo the work of disease or make amends for its ravages. A jaw which has lost the best part of its function with its teeth, or which bites unequally with the scattered survivors of its former armament, is but a deceitful guardian of the passage to the stomach; while it seems to do duty in mastication, it passes intact much that is unfit for the immediate action of the gastric juice. Were the relationship between bad teeth and dyspepsia, with its consequent discomforts, better understood, we should probably hear less of the prevalence of dental caries. Greater attention would then be paid to the small organs whose obscure influence on the general health so fully justifies their preservation. Specific constitutional disease, drugs, and other special factors no doubt account for a certain amount of dental decay. Neglect, however, accounts for much more. Want of care in choosing food, and particularly in cleansing the teeth, has nearly everything to do with the dental worries of a great many people. A point well worthy of note in this connection is that most of the permanent set of teeth come into active operation during childhood or early youth. It is hardly to be expected that children, if let alone, will pay much attention to the state of their mouths, unless driven to notice an aching stump. Thus it happens that most children have lost one or several teeth before they are well into their teens. Here, then, there is need for parental supervision. Mr. W. M. Fisher, of Dundee, has been led by certain observations, which proved the defective condition of the teeth in a majority of school children, to suggest that some regular system of supervision by a dentist be adopted as a part of school management. The expenses he would have defrayed by the parents, or, should they be too poor, by the State, out of the education grant. This plan has actually been adopted in the parochial school at Anerley, in Surrey. We have long been of opinion that it would be most desirable if the health of children in all schools could by some plan be periodically passed under review by a medical inspector. The possible obstacle to such an arrangement would be the expense. This may not prove insuperable, and if it does not, we may hope that this method, and also some plan of dental supervision, may find their place among the recognized forms of school discipline.

